# Mantle cell lymphoma presenting with lethal atraumatic splenic rupture

**DOI:** 10.4322/acr.2021.340

**Published:** 2021-11-05

**Authors:** Frederick Eyerer, Juli-Anne Gardner, Katherine A. Devitt

**Affiliations:** 1 University of Vermont Medical Center, Department of Pathology and Laboratory Medicine, Burlington, VT, USA; 2 University of Vermont, Larner College of Medicine, Burlington, VT, USA

**Keywords:** Lymphoma, Mantle-Cell, Splenic Rupture, Cyclin D, Lymphoma, Malignant

## Abstract

Mantle cell lymphoma is characterized by t(11;14) with *CCND1-IGH* fusion and manifests with a spectrum of disease ranging from relatively indolent to aggressive. Here, we present a case of pleomorphic mantle cell lymphoma with three fusion signals that presented with lethal atraumatic splenic rupture. We discuss on the implications of variant CCND1 signal patterns as well as the epidemiology and pathophysiology of atraumatic splenic rupture.

## INTRODUCTION

Mantle cell lymphoma (MCL) is B-cell neoplasm with a spectrum of clinical manifestations ranging from indolent to aggressive disease. It represents 3-10% of newly diagnosed cases of non-Hodgkin lymphoma in Western countries and predominantly affects men (M:F 2:1) with a median age of 60 years.^1^^-^3 No clear risk factors have been identified.^1^ Despite improved understanding of its pathogenesis over the past 30 years, treatment options and overall survival have remained poor.^2^^,^^4^

Greater than 95% of cases of MCL are characterized by gene fusion of *CCND1*, the Cyclin D1 gene on chromosome 11, with *IGH*, the immunoglobulin heavy chain gene on chromosome 14. The remaining cases often demonstrate fusions with the Cyclin D2 or Cyclin D3 gene.^3^^,^^5^^,^^6^ By immunohistochemistry, more than 95% of cases demonstrate Cyclin D1 overexpression, and SOX-11 has greater than 90% sensitivity, including the cases that are Cyclin D1-negative.^3^^,^^7^^,^^8^ Diagnosis is often guided by the use of flow cytometry, which classically demonstrates positivity for CD5, bright CD20, and FMC7 and negativity for CD23 and CD10.^3^

Although the diagnosis of MCL carries a poor prognosis with a median survival of 3-5 years, it can be further subclassified into several morphologic variants that have a range of prognoses.^9^^,^^10^ The most indolent is leukemic non-nodal MCL, which has a median survival of 79 months.^11^ Classic MCL has an intermediate survival, typically closer to a median of 5 years and is morphologically characterized by a population of small to medium cells with variably irregular nuclear contours.^3^^,^^12^ The blastoid and pleomorphic variants, characterized by their name-sake morphologies, portend the worst prognosis, with a median survival of 14.5 months.^12^^,^^13^ Predicted survival for MCL can be stratified by the Mantle Cell International Prognostic Index (MIPI), which is calculated based on age, ECOG performance status, LDH levels, and WBC counts.^14^ Ki-67 index has also shown to be an independent prognostic factor and captures the impact of blastoid and pleomorphic morphologies on prognosis.^15^

MCL presents with advanced stage disease (stage III-IV) in 85-87% of cases.^16^^,^^17^ Roughly 14-25% of patients present with “B–symptoms” of fever, fatigue, malaise, night sweats.^16^^,^^17^ The lymph nodes are the primary site of involvement in 75% of cases, with diffuse and localized lymph node involvement present in 57% and 30% of cases, respectively.^16^^,^^17^ Extranodal sites of involvement include the bone marrow (69-79%), spleen (47%), peripheral blood (36%), gastrointestinal tract (18%), colon (13%), liver (13%), head and neck (12%), and less frequently in other sites including the pleura, lung, skin, and cerebrospinal fluid.^16^^,^^18^

Herein, we present a case of pleomorphic MCL that presented with lethal atraumatic splenic rupture which has only rarely been reported in the literature.

## CASE REPORT

**Figure 1:**
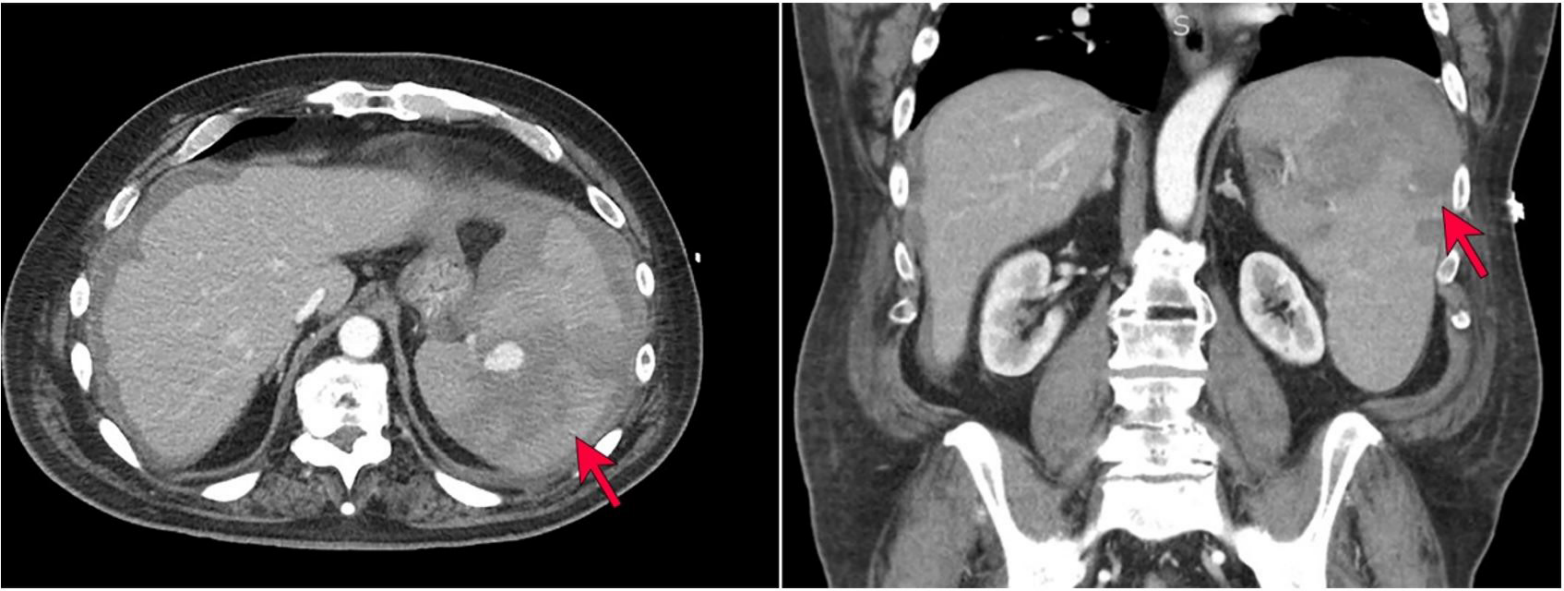
CT imaging demonstrating splenomegaly with hemorrhage, splenic laceration, and perisplenic hematoma (red arrows). Lymphadenopathy was also noted on imaging (not shown).

The patient is a 70-year-old man with a past medical history of hypertension, insulin-dependent diabetes mellitus, meningioma status post resection, and colon cancer status post resection who presented to an outside hospital with acute onset abdominal pain and was found to have a 10 cm spontaneous grade IV splenic laceration with active extravasation (Figure 1) splenomegaly, and external iliac lymphadenopathy (4.1cm in greatest dimension)..

During transfer to our tertiary care hospital, he had increased abdominal distension and became hypotensive. He was given two units of packed red blood cells but was hemodynamically unstable and became unresponsive upon arrival. A massive transfusion protocol was initiated with temporary regain of consciousness. An emergent IR coil embolization was performed and the patient was transferred to the surgical ICU. Over the next few hours, despite supportive measures, he became progressively hypotensive, acidotic, and hypercarbic. The patient expressed that he wished to discontinue treatment and die peacefully. His family was brought to the bedside and the patient was moved to comfort-measures only. He passed away shortly thereafter. An autopsy was not performed

**Figure 2:**
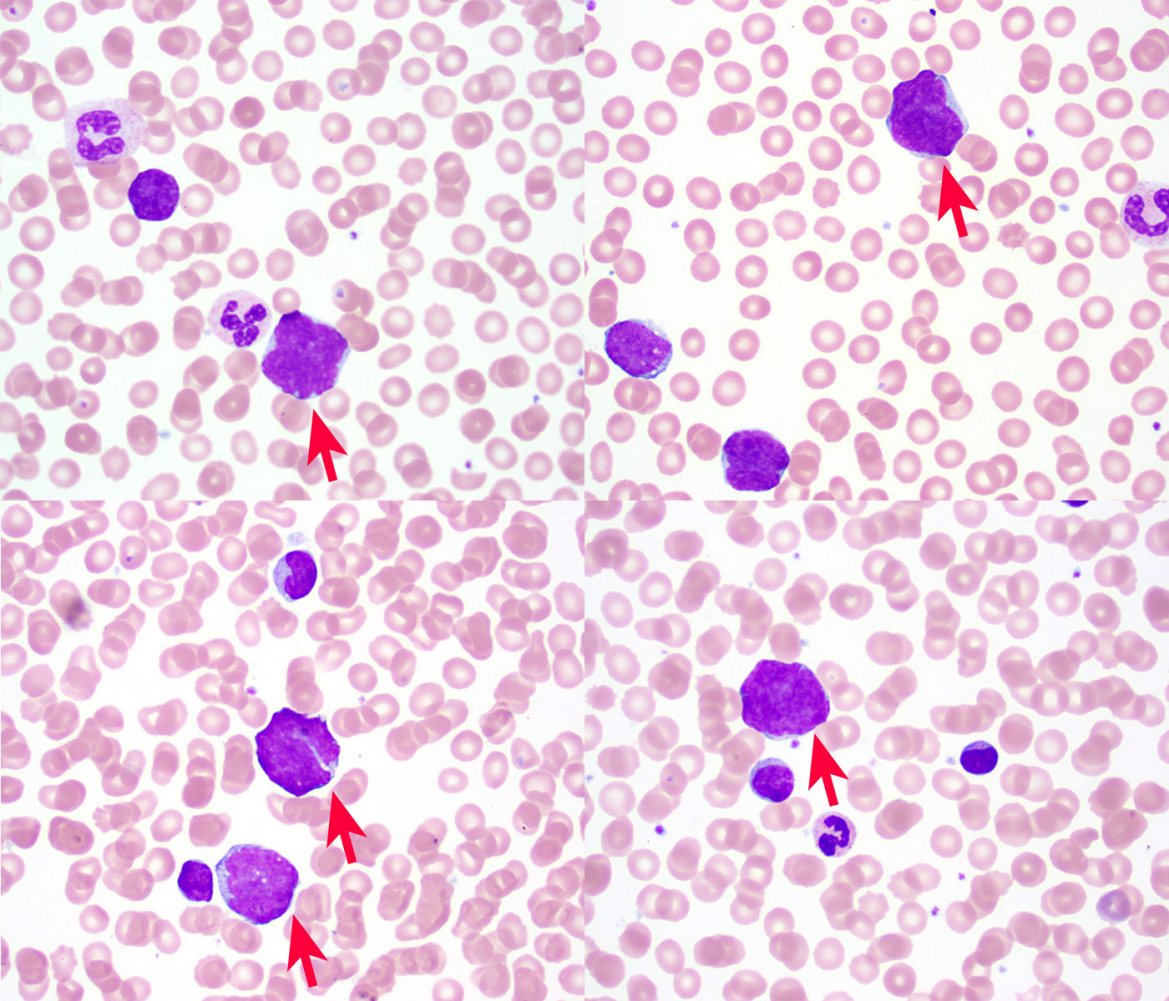
Peripheral blood smear (Wright Giemsa, 100X) demonstrating pleomorphic atypical lymphocytes (red arrows).

Complete blood count on arrival showed a white blood cell count of 42,020/mm^3^ (Reference range [RR]; 4,000-10,400/mm^3^) with 79% lymphocytes, 11% monocytes, 10% neutrophils, red blood cell 3.49 M mm^3^ (RR; 4.46-5.78 M/mm^3^), hemoglobin 10.0 g/dL (RR; 13.8-17.3 g/dL), hematocrit 31.0% (RR; 39.5-50.2%), MCV 89 fl (RR; 81-95/fl), platelet 116,000/mm^3^ (RR; 141,000 – 377,000/mm^3^). Examination of the peripheral smear showed nucleated red blood cells and a population of atypical lymphocytic cells characterized by variable size, irregular nuclear contours, and high nuclear:cytoplasmic ratios (Figure 2).

**Figure 3:**
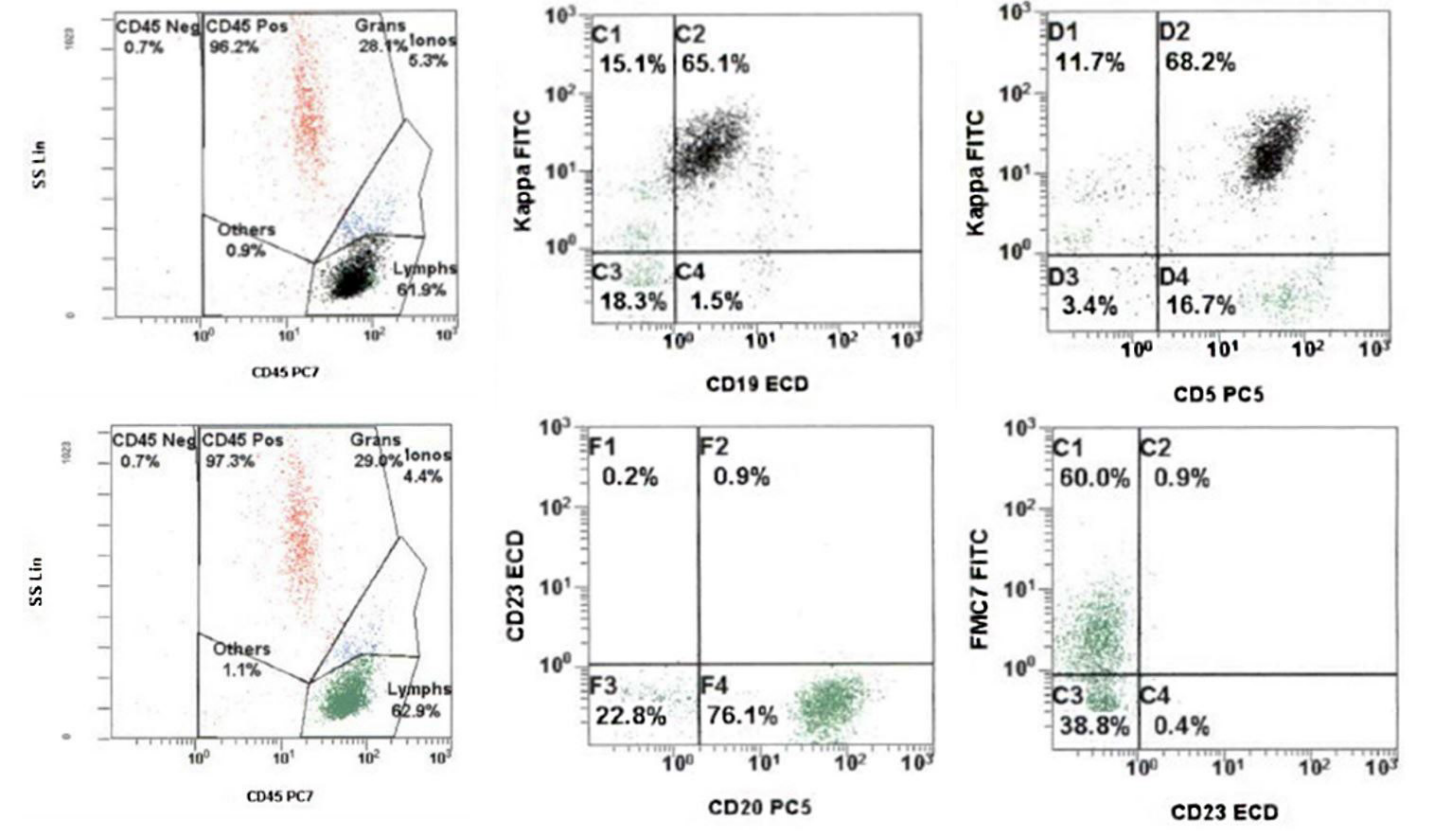
Flow cytometric analysis showing a clonal population of kappa-restricted CD5+ B-cells that express FMC7 and bright CD20 and lack CD23 (Top row: black dots backgated on CD19+ events).

Flow cytometry revealed a clonal population of kappa-restricted B-cells that were CD19+, CD5+, CD23-, FMC7+, and bright CD20+ (Figure 3).

**Figure 4:**
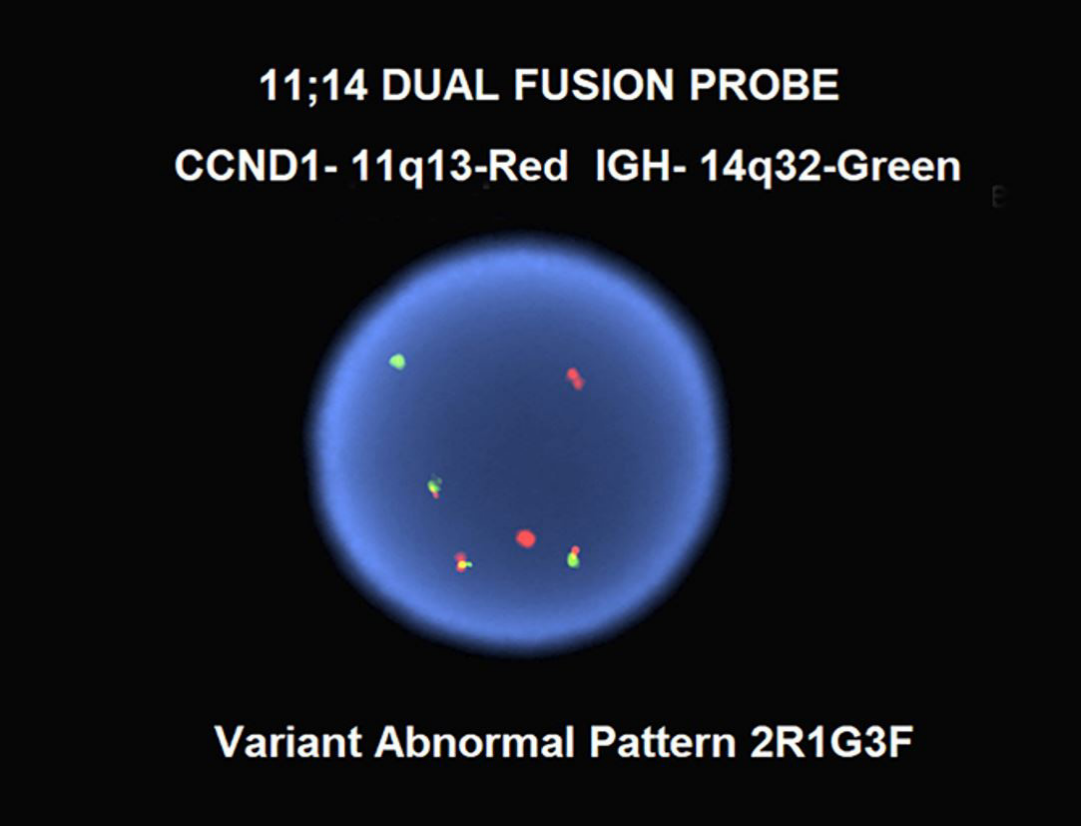
Dual fusion FISH for CCND1 and IGH, demonstrating a variant abnormal signal pattern with three copies of CCND1-IGH fusion (yellow signals).

This immunoprofile is typical of mantle cell lymphoma. This diagnosis was confirmed by fluorescence in situ hybridization (FISH) analysis which was positive for t(11;14)(q13;q32), demonstrating a variant abnormal pattern with three CCND1-IGH fusion signals (Figure 4).

## DISCUSSION

In a systematic review identifying 845 cases of atraumatic splenic rupture (ASR), Renzulli et al.^19^ identified six major etiological groups: neoplastic (30.3%), infectious (27.3%), inflammatory non-infectious (20.0%), drug- and treatment-related (9.2%), mechanical (6.8%), and normal spleen (6.4%). The mortality rate in this study was 12.2%, with significant risk factors for mortality identified as splenomegaly, age greater than 40 years, and neoplastic disorders. 152 cases of malignant hematological disorders were identified (16.4% of total; 54% of neoplastic etiologies) and included (n, % of malignant hematological disorders): non-Hodgkin lymphomas (55, 36%), Hodgkin lymphoma (4, 3%), acute lymphoblastic leukemia (12, 8%), other leukemias (24, 16%), acute myelogenous leukemia (21, 14%), myeloproliferative disorders (24, 16%), and myelodysplastic disorders (12, 8%).^19^ Of the reported cases of non-Hodgkin lymphoma, at least six (6/55, 11%) of these were mantle cell lymphoma, including five with blastoid morphology and the sixth with an aggressive clinical course.^19^^-^24 With a reported mortality rate of 12.2% in cases of ASR, identifying a specific set of risk factors for ASR in patients with splenomegaly could help inform clinical management. However, there is a paucity of robust data regarding such risk factors in the literature.

The diagnosis of the pleomorphic variant of classic MCL in this case is supported by the finding of pleomorphic lymphoma cells in the patient’s peripheral blood, splenomegaly (20cm) and iliac lymphadenopathy (4.1cm) on imaging, immunophenotype and cytogenetic findings typical of MCL. Biopsies of the spleen and lymph node were not performed. Leukemic non-nodal MCL was ruled out given significant adenopathy. Though atypical t(11;14) presentations may occur in up to 17% of MCL,^25^ detection of three copies of the *CCND1-IGH* fusion gene appears to be associated with the more aggressive variants of MCL. In a case series of leukemic MCL, Rahman et al.^26^ previously reported two cases of large cell leukemic MCL, both of which had three copies of IGH-CCND1, while the remaining 10 cases for which FISH was available lacked both large cell morphology and extra fusion copies. Gruszka-Westwood et al.^27^ reported a case of leukemic MCL with a complex karyotype and multiple copies of the *CCND1-IGH* fusion gene, massive splenomegaly, and an aggressive clinical course. Miao et al.^28^ reported another case of pleomorphic MCL with complex cytogenetics and amplification of the *CCND1-IGH* fusion gene that initially presented as leukemic MCL (classic variant) and later transformed to the pleomorphic variant. As discussed by Miao et al.,^28^ the impact that the *CCND1-IGH* fusion amplification alone has on the disease course is often masked in these cases by a complex karyotype, which is known to portend an aggressive clinical course. Finally, as reported by Ott et al.,^29^ the pleomorphic variant of MCL often shows tetraploidy which would result in extra copies of the *CCND1-IGH* fusion. Although analysis of the current case is limited by incomplete diagnostic data (karyotype was not performed), the pleomorphic morphology of the leukemic MCL fits into the same category as these previously reported cases. It is unclear whether the more aggressive course seen in pleomorphic MCL can be attributed specifically to the extra copies of *CCND1-IGH* or if this may be just a representation of a complex disease karyotype.

ASR in the setting of splenic involvement by an aggressive lymphoma raises the question of whether the neoplasm’s high proliferative rate had an impact on the risk of splenic rupture, i.e., whether the rate of splenic expansion outpaced the rate of capsular compensation such that the tensile forces were too great to maintain integrity of the capsule. This mechanism as well as two other possible mechanisms of ASR have been proposed: acute splenic compression by abdominal musculature during physiological activities, and progressive subcapsular hemorrhage in the setting of splenic thrombosis or infarction due to reticular endothelial hyperplasia.^30^^-^32 Further elucidating the impact that the first of these mechanisms (atraumatic rupture caused by high proliferation/sequestration) has on increasing the risk of splenic rupture could be clinically useful in management decisions, especially if a subset of hematopoietic neoplasms with rapidly increasing spleen size are identified to be at high risk of rupture. But, in practice, the usefulness of this knowledge is limited by other considerations, such as the morbidity and mortality of a splenectomy procedure and the potentially more pressing issue of starting treatment. Current NCCN guidelines for B-cell lymphomas only include splenectomy as an indication for splenic marginal zone lymphoma.^33^

This case is not the first reported case of ASR in mantle cell lymphoma, nor is it the only pleomorphic/blastoid MCL that presented in the spleen.^20^^-^24^,^^34^ However, to our knowledge it is the first reported case of pleomorphic/blastoid MCL that presenting with lethal ASR. The findings in this case shed further light on the spectrum of disease and clinical sequelae in this uncommon variant of mantle cell lymphoma.

## CONCLUSION

Understanding the structural and physiologic consequences of hematopoietic neoplasms is important for informing screening and treatment decisions. This case of pleomorphic mantle cell lymphoma presented with splenomegaly and lethal atraumatic splenic rupture. While this is a rare presentation of MCL, this case highlights the importance of recognizing the risks of splenomegaly in aggressive lymphomas.
